# Omilancor mitigates the senescence of nucleus pulposus cells induced by DDP through targeting MAP2K6

**DOI:** 10.18632/aging.205588

**Published:** 2024-03-20

**Authors:** Fang Yafeng, Shi Xinpeng, Wei Rong, Cui Guofeng

**Affiliations:** 1 Luoyang Central Hospital Affiliated to Zhengzhou University, Luoyang, Henan, China

**Keywords:** intervertebral disc degeneration, Omilancor, cellular senescence, MAP2K6

## Abstract

Purpose: This study explores the potential of Omilancor in treating Intervertebral Disc Degeneration (IDD) through MAP2K6 targeting.

Methods: We analyzed mRNA microarray datasets to pinpoint MAP2K6 as a key regulator implicated in IDD progression. Follow-up studies demonstrated that cisplatin (DDP) could prompt cellular senescence *in vitro* by upregulating MAP2K6 expression. Through molecular docking and other analyses, we identified Omilancor as a compound capable of binding to MAP2K6. This interaction effectively impeded the cellular senescence induced by DDP.

Results: We further showed that administration of Omilancor could significantly alleviate the degeneration of IVDs in annulus fibrosus puncture-induced rat model.

Conclusions: Omilancor shows promise as a treatment for IDD by targeting MAP2K6-mediated cellular senescence.

## INTRODUCTION

Low back pain (LBP) is a significant contributor to global disability, placing substantial strain on both the social economy and healthcare systems [1, 2]. Studies indicate that over 80% of individuals will encounter LBP lasting more than 4 weeks at some point in their lives, with around 10% eventually becoming disabled [3, 4]. Intervertebral disc degeneration (IDD) is a widely recognized primary factor behind LBP, yet our current grasp of its underlying mechanisms remains inadequate.

The intervertebral disc (IVD) constitutes a distinctive and relatively enclosed tissue structure, characterized by an inner proteoglycan-rich jelly-like nucleus pulposus (NP), encompassed by the outer annulus fibrosus (AF) interwoven by multiple layers of collagen fibers, and the upper and lower cartilaginous endplates (EP) [2, 5]. It is widely acknowledged that dysfunction within the NP tissue plays an important role in the pathological process of IDD [6, 7]. Therefore, increasing the number of functional NP cells has emerged as the key to reestablish the function of IVDs [8].

Numerous studies have shown that inflammation plays a crucial role in driving the progression of IDD. This encompasses processes like extracellular matrix (ECM) degradation, cellular senescence, necrosis, and programmed cell death [9, 10]. Current research in the IVD field predominantly centers around cell apoptosis and pyroptosis, while research on cellular senescence in the progression of IDD remains at an early stage, warranting further comprehensive exploration [11].

Mitogen-activated protein kinase 6 (MAP2K6) belongs to the MAPKK family and is involved in a range of cellular processes, including gene expression, mitosis, metabolism, motility, survival, apoptosis, and differentiation. These functions are achieved through the activation of MAPK activity [12, 13]. There are reports indicating that once MAP2K6 is activated, it triggers the cleavage and subsequent activation of the MAPK signaling pathway, ultimately leading to cellular senescence [14, 15]. The circ_016719/miR-29c axis was identified as a regulator of MAP2K6 in neuronal apoptosis resulting from ischemia/reperfusion (I/R) [16]. In esophageal adenocarcinoma, inhibition of MAP2K6 was found to curtail tumor cell proliferation [17]. Notably, Dan He et al. demonstrated that the deterioration of intestinal function associated with aging is facilitated by the activation of the mTORC1-p38MAPK-p53 pathway within intestinal stem cells. This age-related decline can be mitigated by disrupting either mTORC1 or p38MAPK activity [18]. Yingmin Zhang et al. reported that suppression of the ROS/MAP2K6/p38 signaling pathway could inhibited cell senescence in Human fibroblasts [19]. However, whether the downregulation of MAP2K6 would reduce cellular senescence in NP cells is still unclear.

Omilancor is a locally-acting, first-in-class, oral therapeutic, and is widely used in clinical practice [20]. Mechanistically, Omilancor is the lead agonist of Lanthionine Synthetase C-like 2 (LANCL2) pathways, a validated therapeutic target for autoimmune, metabolic, and infectious diseases [21, 22]. But research in the field of the correlation between Omilancor and cellular senescence is still in its infancy.

The above-mentioned studies and our preliminary experimental results indicated that MAP2K6-mediated cellular senescence may play an important role in IDD, but its detailed mechanism is still unclear.

## RESULTS

### MAP2K6 exhibited heightened expression levels in IDD

**Figure 1:**
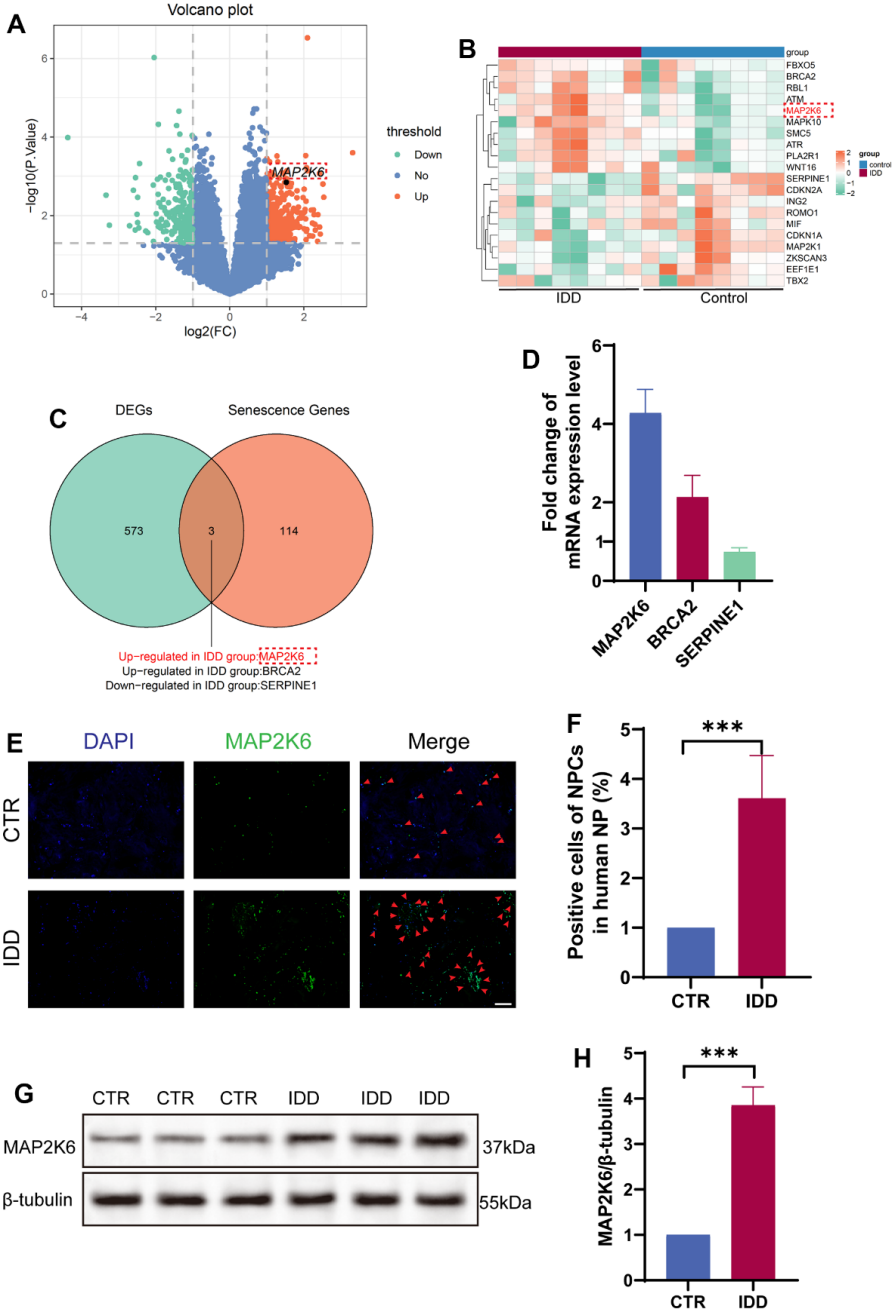
**MAP2K6 exhibited heightened expression levels in IDD.** (**A**) Volcano plot illustrating the differential gene expression in human NP samples Note: GSE186542, GSE185728, GSE167199, (Control group [CTR]: n = 8, Intervertebral Disc degeneration [IDD]: n = 8). (**B**) Heatmap depicting the top 20 differentially expressed genes (DEGs) in human NP. (**C**) Venn diagram illustrating the intersection of DEGs between the CTR and IDD groups and senescence-related genes. (**D**) RT-qPCR results presenting the expression levels of candidate genes (MAP2K6, BRCA2, and SERPINE1) in degenerative NP cells. (**E**, **F**) Immunofluorescence staining (IF) and corresponding quantitative analysis of MAP2K6 in CTR and IDD human NP samples. (Original magnification 100×, scale bar = 400 μm; Red arrow: positive cells). (**G**, **H**) Western Blot analysis and quantitative assessment of MAP2K6 expression in human NP samples. CTR, control group; IDD, intervertebral disc degeneration group. n = 3 each group. Data are represented as mean ± standard deviation. ***p < 0.001, as determined by t-test.

A comprehensive assessment was undertaken by analysing a collective compilation of three mRNA microarray datasets (including 8 healthy controls and 8 IDD patients) sourced from the Gene Expression Omnibus (GEO) repository to unearth the differentially expressed genes (DEGs) in IDD. A mean fold change >5 or <0.2 and p-values <0.01 were taken as criteria. As a result, 627 DEGs including 398 upregulated and 229 downregulated DEGs were identified in IDD samples compared with healthy controls (Figure 1A, 1B). And then, to unravel the underlying roles of DEGs in cellular senescence, these DEGs were intersected with senescence-related genes cataloged in KEGG database (Figure 1C). To substantiate these findings, 2 upregulated genes (MAP2K6, BRCA2) and 1 downregulated gene (SERPINE1) were examined by qRT-PCR analysis employing an independent cohort encompassing 3 IDD patients and 3 controls. MAP2K6 was found to be significantly upregulated in nucleus pulposus (NP) tissues extracted from IDD patients in contrast to those obtained from control patients (Figure 1D). This observation was further corroborated by immunofluorescence (IF), western blot and quantitative analyses (Figure 1E–1H). Therefore, we selected MAP2K6 for further investigation. These data collectively suggest a potential pivotal role for MAP2K6 in the progression of IDD.

### MAP2K6 was upregulated in DDP-induced cellular senescence model

**Figure 2:**
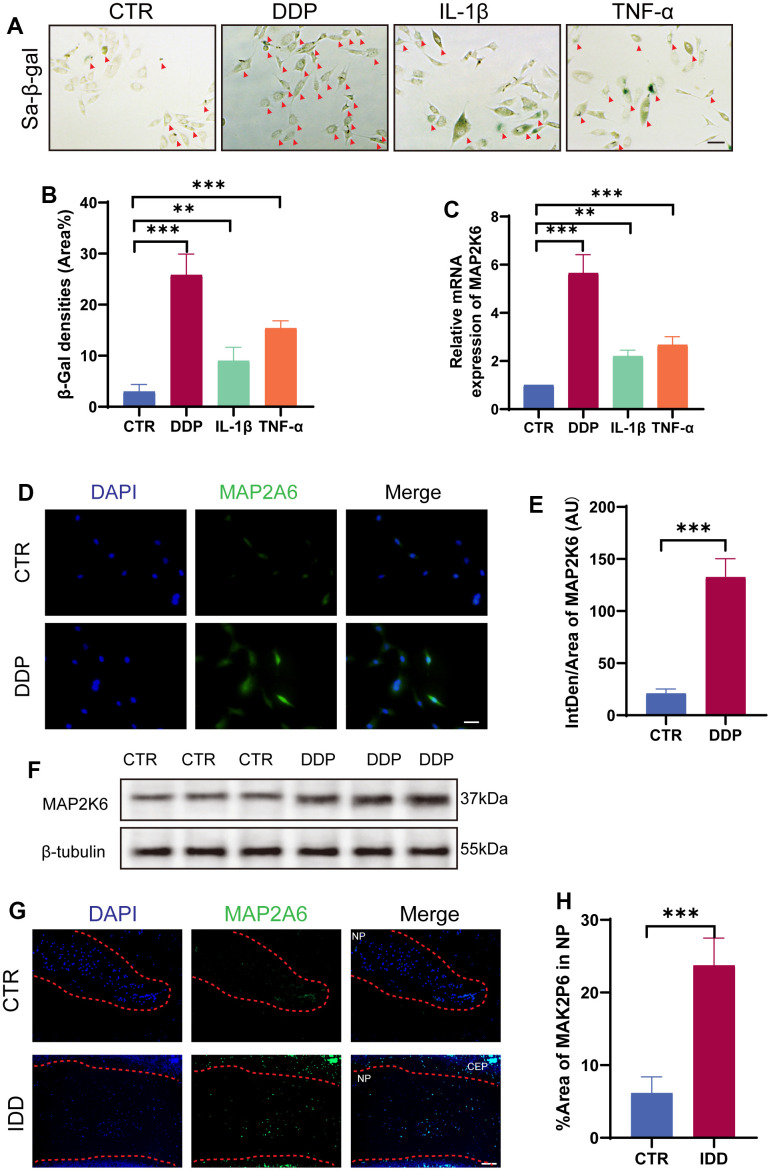
**MAP2K6 was upregulated in DDP-induced cellular senescence model.** (**A**, **B**) Rat NP cells were exposed to DDP (mM, 24h), IL-1β (100ng/ml, 24h), and TNF-α (50ng/ml) to establish an *in vitro* senescence NP cell model, which was confirmed by SA-β-gal staining and the corresponding quantitative analysis. (Original magnification 200×, scale bar = 200 μm; Red arrow: positive cells). (**C**) RT-qPCR results depict the expression levels of MAP2K6 following different treatments. (**D**, **E**) Immunocytochemistry (ICC) staining and the accompanying quantitative analysis of MAP2K6 in control (CTR) or DDP-treated Rat NP cell models. (Original magnification 400×, scale bar = 100 μm). (**F**) Western Blot analysis illustrating the expression of MAP2K6 in control (CTR) or DDP-treated Rat NP cell models. (**G**, **H**) Immunofluorescence (IF) staining of IVDs tissue in rat model and quantitative assessment of MAP2K6 expression in control (CTR) or IDD rats after 8 weeks of operation. (original magnification 400×, scale bar = 100 μm). CTR, control group; DDP, cisplatin treatment. n = 3 each group. Data are represented as mean ± standard deviation. ns: not significant, *p < 0.05, **p < 0.01, ***p < 0.001, as determined by t-test.

IL-1β and TNF-α are cytokines intricately involved in the pathogenesis of IDD, and frequently harnessed to establish an *in vitro* cellular model simulating the degenerative process. Cisplatin (DDP), known to initiate the DNA damage signaling cascade and activate sensory proteins triggering cellular senescence, has been widely applied in the establishment of cellular senescence models. Therefore, we first detected SA-β-gal activity to evaluate abovementioned modeling methods and found out that DDP was the ideal treatment for the construction of a robust cellular senescence model (Figure 2A, 2B). Subsequently, to investigate the role of MAP2K6 in the pathogenesis of IDD, qRT-PCR was used to ascertain the expression level of MAP2K6 within our cellular senescence models. Remarkably, our findings showed an upsurge in MAP2K6 expression across all cellular models, especially in DDP-induced cellular senescence model (Figure 2C). The veracity of this finding was further buttressed through IF and quantitative analyses (Figure. 2D–2F).

To investigate the role of MAP2K6 in the progression of IDD *in vivo*, we resorted to annulus fibrosus (AF) puncture-induced IDD rat model and found out that the expression level of MAP2K6 was significantly increased in the IDD group (Figure 2G, 2H). In summary, these findings underscore the plausible centrality of MAP2K6 in cellular senescence and IDD.

### Omilancor reduced cellular senescence by regulating MAP2K6

**Figure 3:**
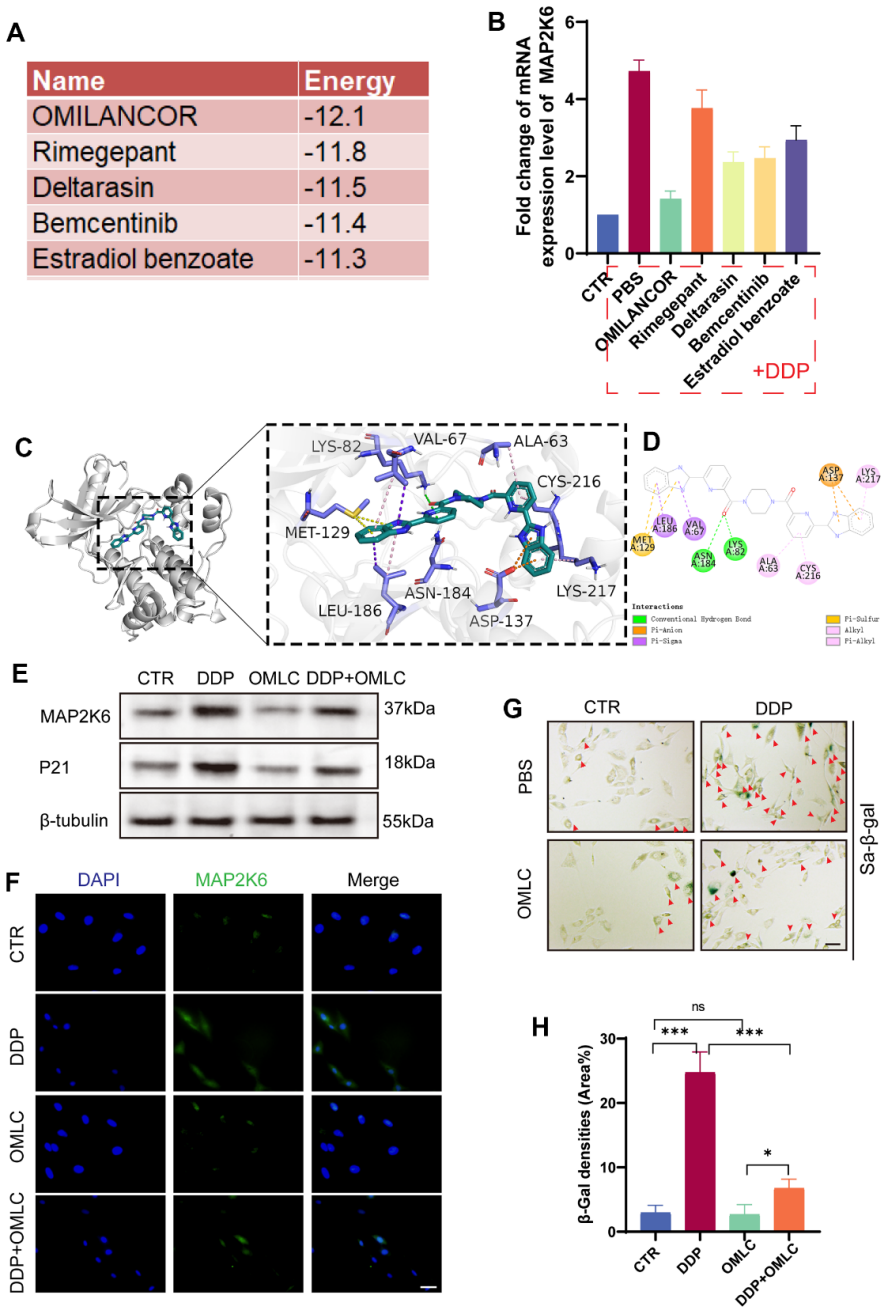
**Omilancor reduced cellular senescence by regulating MAP2K6.** (**A**) The top 5 drugs and their corresponding binding energies (kcal/mol) predicted as potential inhibitors for MAP2K6. (**B**) RT-qPCR results revealing that Omilancor exhibited the most significant inhibitory effect on MAP2K6 expression (5ug/ml, 12h, n=3 each group). (**C**, **D**) Ligand interaction diagrams depicting the top-scoring molecular docking complexes between OMLC and MAP2K6 proteins (RMSD = 2.206; estimated free energy of binding = -12.1 kcal/mol). (**E**) Western blot analysis illustrating the expression levels of MAP2K6 and P21 following treatments with DDP and OMLC. (**F**) Immunocytochemistry (ICC) staining demonstrating the expression of MAP2K6 in rat NP cells subjected to various treatments. (Original magnification 400×, scale bar = 100 μm). (**G**, **H**) SA-β-gal staining and the corresponding quantitative analysis of rat NP cells under different treatment conditions. (Original magnification 100×, scale bar = 400 μm). Red arrow: positive cells). CTR, control group; DDP, cisplatin treatment; OMLC, Omilancor treatment. n = 3 each group. Data are represented as mean ± standard deviation. ns: not significant, *p < 0.05, **p < 0.01, ***p < 0.001, as determined by one-way ANOVA with post-hoc Bonferroni correction or Kruskal-Wallis H test with a Dunn’s correction.

Subsequently, we embarked on an investigation for the potential of a drug or compound to alleviate IDD by regulating MAP2K6-mediated cellular senescence. AlphaFold2 software was used to predict the structure of MAP2K6 and assess its reliability. Then, we used a natural drug library of 4948 compounds to screen out the ones with good docking with MAP2K6, docking prediction analysis showed that several compounds from the ligand library exhibited a great capability to interact with MAP2K6 (Figure 3A). In alignment with the predicted free energy of binding results, qRT-PCR analysis demonstrated that Omilancor significantly downregulated the expression of MAP2K6 in NP cells treated with DDP (Figure 3B). Subsequently, we predicted and visualized the binding sites and druggable sites by AutoLigand and PyMOL (Figure 3C, 3D).

To validate its potential in attenuating cellular senescence *in vitro*, we employed Omilancor as a pre-treatment for NP cells followed by subsequent DDP treatment. Western blot analysis suggested that pre-treatment with Omilancor significantly reduced the expression levels of MAP2K6 and p21, a recognized inhibitor of the cell cycle, compared to NP cells treated with DDP alone (Figure 3E). This result was further substantiated by subsequent IF experiments (Figure 3F). The SA-β-gal analysis revealed that pre-treatment with Omilancor notably inhibited DDP-induced cellular senescence *in vitro* (Figure 3G, 3H). Taken together, these results indicated that Omilancor mitigated cellular senescence, thereby alleviating IDD by downregulating the expression of MAP2K6 *in vitro*.

### Omilancor alleviated senescence mediated IDD *in vivo*

**Figure 4:**
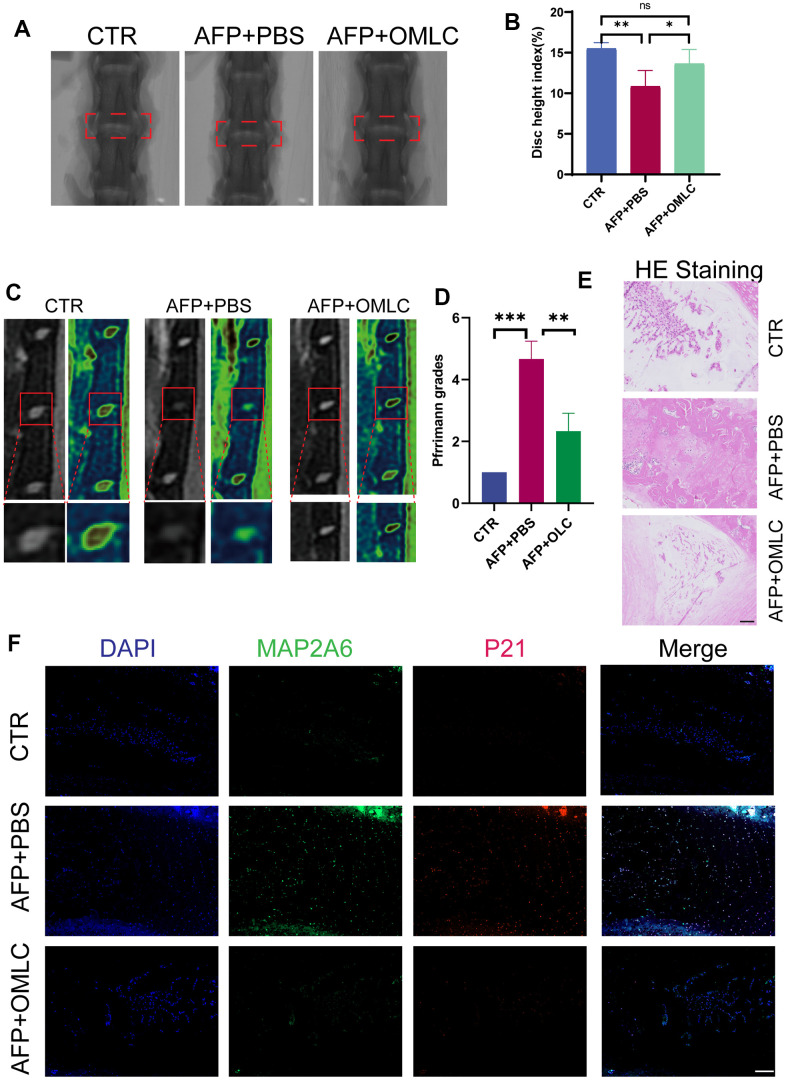
**Omilancor alleviated senescence mediated IDD *in vivo*.** (**A**, **B**) X-ray images and disc height analysis of rats from the CTR, AFP plus PBS, and AFP plus OMLC groups, taken 8 weeks post-operation. (**C**, **D**) MRI images and Pfirrmann grade analysis of rats from the CTR, AFP plus PBS, and AFP plus OMLC groups, obtained 8 weeks after surgery. (**E**, **F**) H&E and IF staining depicting MAP2K6 and P21 in rats from the CTR, AFP plus PBS, and AFP plus OMLC groups, conducted 8 weeks post-operation. (Original magnification 200×, scale bar = 200 μm). CTR, control group; AFP, annulus fibrosus puncture group; n = 3 each group. Data are represented as mean ± standard deviation. ns: not significant, *p < 0.05, **p < 0.01, ***p < 0.001, as determined by one-way ANOVA with post-hoc Bonferroni correction or Kruskal-Wallis H test with a Dunn’s correction.

To elucidate whether Omilancor could act as an effective therapeutic agent for IDD *in vivo*, we introduced Omilancor to the AF puncture-induced IDD rat model. Omilancor or PBS was locally injected into the punctured intervertebral disc during the surgical procedure. Subsequent to an 8-week observation period, radiological examination results indicated that locally administered Omilancor significantly alleviated the degeneration of IVDs (Figure 4A–4D), this encouraging radiological findings were further substantiated by histological assessments using H&E staining (Figure 4E). Moreover, IF results demonstrated that the expression levels of both MAP2K6 and P21 were significantly decreased in the Omilancor-treated group compared with the control or the PBS-administered group (Figure 4F).

These results collectively indicated that Omilancor could impede the progression of cellular senescence by suppressing the expression of MAP2K6. This nuanced modulation, in turn, exerts a mitigating influence on IDD. Consequently, these findings supported the hypothesis that regulating MAP2K6 is a promising strategy for biological therapy of IDD.

## DISCUSSION

Cellular senescence holds a significant role in the progression of IDD [8, 23]. In this study, we verified that deactivation of MAP2K6 could hinder NP cells senescence induced by DDP. *In vivo*, utilizing an AF puncture-induced IDD rat model, we validated that inactivating MAP2K6 by Omilancor treatment aids in avoiding the reduction of IVDs height, loss of NP water content, loss of NP cells and degradation of ECM caused by AF puncture. This experiment indicates the potential viability of MAP2K6 as a key molecular target for attenuating or potentially reversing IDD.

Cellular senescence refers to the progressive decline in cell proliferation, differentiation capabilities, and physiological functions over time [24]. More importantly, senescence contributes to cell cycle arrest and ECM degradation in NP cells, suggesting that countering senescence is also a key aspect in alleviating the progression of IDD [25]. During the progression of IDD, NP cells senescence escalates, thereby stimulating the release of senescence-associated secretory phenotype (SASP) [26]. This gives rise to an inflammatory microenvironment, further intensifying the senescence process in adjacent cells [27, 28].

MAPK pathways serve as crucial mediators of many biological processes, including cell growth and apoptosis [29]. At the heart of each MAPK pathway lies a cascade comprising three kinases: MAPK, MAPKK and MAPKKK. While extensive studies have been dedicated to elucidating the MAPK proteins-mediated molecular mechanism such as Erk1/2, Jnk1/2/3, P38, there remains a scarcity of reports focusing on the role of MAPKK proteins in the progression of IDD [30–32]. Here we found that the expression of MAP2K6 was significantly increased in degenerated IVDs from IDD patients, indicating a positive correlation between the expression level of MAP2K6 and the degree of IDD.

Cellular senescence was historically categorized into two types: replicative senescence (RS) regulated by the p53-p21-RB pathway in a telomere-dependent manner, and stress-induced premature senescence (SIPS), which activates the p16^INK4a^-RB pathway independently of telomere length. Recent studies demonstrated that inhibiting p53-p21-RB pathway could alleviate senescence-induced IDD [33, 34]. We verified that DDP could induce NP cells senescence *in vitro* via well-established cell models. And then we detected increased expression of MAP2K6 both in cell models and surgically induced IDD rat model, suggesting that MAP2K6 may be involved in the progression of IDD by regulating cellular senescence.

An increasing number of researches are exploring the use of ligands in IDD animal models to downregulate specific genes or deactivate proteins in NP tissues [35–37]. This study predicted the capacity of Omilancor in binding MAP2K6, subsequently substantiated that it could downregulate the expression of MAP2K6 in DDP-induced NP cell senescence model with following cellular experiments. To verify the therapeutic role of Omilancor in alleviating degeneration of IVDs *in vivo*. We administrated Omilancor to an AF puncture-induced IDD rat model, and found that Omilancor treatment significantly lightened the degeneration of IVDs, with higher signal of T2-weighted MRI images and histological scores. Consistently, the expression levels of MAP2K6 and p21 were also reduced in the Omilancor-treatment group. These exciting findings showed that downregulation of MAP2K6 could repress the progression of IDD, and supported our hypothesis that regulating MAP2K6 is a promising strategy for biological therapy of IDD.

However, our study also has limitations. We emphasized the role of MAP2K6/p21 axis in DDP-induced NP cells senescence, but whether p21 was activated by MAP2K6 directly was still unclear. Given the potential cytotoxic effects of DDP in cell senescence model, simply inhibiting cellular senescence might not be enough for a satisfactory outcoming. An improved and more physiologically relevant cellular model is urgently needed to be developed. Although the therapeutic effect of Omilancor in the AF puncture-induced IDD rat model was effective, the translation of these findings to clinical applications may face challenges, including the need for further investigations to address safety concerns and potential side effects. Additionally, the specific mechanisms and optimal dosage for clinical use remain areas that require further exploration. There are still several problems to be solved, and many hurdles to overcome before it is administered to humans.

Altogether, our study demonstrated that Omilancor could downregulate active MAP2K6 levels, consequently impeding the senescence process of NP cells and ultimately retarding IDD. Thus, suppressing MAP2K6/p21 axis holds promise as a potentially effective therapeutic approach for addressing IDD.

## MATERIALS AND METHODS

### NP tissues collection

A total of 6 NP samples was obtained from 6 patients (male: female=3: 3) underwent spinal surgery at the Luoyang Central Hospital. The degree of disc degeneration was graded according to the Pfirrmann system, based on T2-weighted images assessed by three independent observers. We collected degenerative NP tissues from disc herniation cases, which were graded as III, IV, or V. Control NP tissues, graded as I or II, were obtained from fresh traumatic lumbar fracture or hemivertebra cases (Table 1 and Supplementary Figure 1A).

**Table 1:** Information of human disc samples from 6 patients.

**Human disc samples**	**Gender**	**BMI**	**Age (y)**	**Diagnosis**	**Level**	**Grade**
1	F	20.7	10y	Hemivertebra	L1/2	I
2	F	21.3	20y	Traumatic	L1/2	I
3	M	24.6	8y	Hemivertebra	L1/2	I
4	F	28.8	41y	Disc herniation	L3/4	IV
5	M	25.8	30y	Disc herniation	L4/5	IV
6	M	29.6	30y	Disc herniation	L3/4	V

### GEO expression datasets and bioinformatics analysis

The Gene Expression Omnibus (GEO) database (https://www.ncbi.nlm.nih.gov/geo/) is an international public repository that distributes high throughput gene expression datasets. To investigate the differences in expression profiling and relevant biological processes in IDD patients, we searched for datasets in the GEO database using the keywords: (1) “intervertebral disc degeneration” [All Fields] AND (2) “Homo sapiens” [porgn] AND (3) “gse” [Filter] AND (4) “2015/01” [Update Date]. The inclusion and exclusion criteria were as follows: datasets should be the whole-genome expression data of mRNA; expression data should be obtained from human annulus disc tissue of IDD patients and healthy controls. Finally, we included a total of three mRNA expression datasets (including GSE186542, GSE185728, GSE167199) in our study. The detail information of above-mentioned datasets was shown in (Table 2 and Supplementary Table 1).

**Table 2:** Information of microarray datasets.

**Dataset**	**Control**	**IVDD**	**Platform**	**Year**	**Country**	**Author**	**PMID**
GSE186542	3	3	GPL24676 Illumina NovaSeq 6000 (Homo sapiens)	2021	China	Wu Y	36746221
GSE185728	2	2	GPL24676 Illumina NovaSeq 6000 (Homo sapiens)	2021	China	Wu J	35619555
GSE167199	3	3	GPL24676 Illumina NovaSeq 6000 (Homo sapiens)	2021	China	Li Z	26484230

Identifying essential cell Senescence regulator genes in IDD. In brief, 114 cell senescence regulator genes were obtained from the CellAge database (https://genomics.senescence.info/cells/). Finally, we took an intersection of the differentially expressed genes in IDD and cell senescence regulator genes and got a total of 3 essential cell senescence regulator genes (MAP2K6, BRCA2, and SERPINE1) in IDD for further analysis. The expression datasets were analyzed by the R software version 4.0.0 (http://www.r-project.org). After quantile normalization of the raw data, we identified differentially expressed mRNAs with statistical significance between the two groups using Volcano Plot and Heat Map filtering.

### qRT-PCR

Total RNA extraction from NP tissue or cultured NP cells was performed with TRIzol Reagent (Ambion, Life Technologies, Carlsbad, CA, USA) according to the manufacturer’s instructions. A Nanodrop (Thermo Fisher Scientific, USA) was used to analyze the RNA quantity. Then, the mRNA was converted to complementary DNA (cDNA) using a reverse transcription kit (Takara, Cat. #RR047A, Japan). Relative expression levels were analyzed using the comparative Ct (ΔΔCT) method (2^−ΔΔCT^ logarithm transformed). GAPDH was used to normalize mRNA expression level. The following primers were used: human MAP2K6 (F: 5’-GCCACAGTTAATAGCCAGGAA-3’, R: 5’-GCCCACATATCCAGAGCTTAT-3’), human BRCA2 (F: 5’-CGGATGGAAGAGAGACTCAAA-3’, R: 5’-ACAGGAAATTGAACAGCGATT-3’); human SERPINE1 (F: 5’-CAGTGGCTACCTGGTCGACGAAGTTGCTAAAGAGATCGATGCCGGTTGCAAACCATAC-3’); human MAP2K6 (R: 5’-GTATGGTTTGCAACCGGCATCGATCTCTTTAGCAACTTCGTCGACCAGGTAGCCACTG-3’); and human GAPDH (F: 5’-GACAGTCAGCCGCATCTTCTT-3', R: 5'-AATCCGTTGACTCCGACCTTC-3').

### Western blot analysis

Total protein from NP tissue or cultured NP cells was extracted using RIPA lysis buffer supplemented with protease inhibitors cocktail (Thermo Fisher Scientific, USA). The protein concentrations were detected using a BCA Protein Assay Reagent Kit (KeyGEN BioTECH, China) according to the manufacturer’s instructions. Then, the lysates were separated on 8-12% sodium dodecyl sulphate-polyacrylamide gels (Epizyme Biotech, China), and transferred to 0.22μm polyvinylidene fluoride (PVDF) membranes (Millipore, USA). Membranes were blocked for 2 h in TBS-T with 5% bovine serum albumin (BSA), and incubated with primary antibodies (MAP2K6, diluted 1:5000, Cat. #12280, Cell Signaling Technology, USA; p21, diluted 1:5000, Cat. #ab109199, Abcam, USA; β-Tubulin, diluted 1:1000, Cat. #5666, Cell Signaling Technology, USA) at 4° C overnight. After being washed with TBS-T, the membranes were incubated with HRP-linked anti-rabbit immunoglobulin G (IgG; diluted 1:5000, Cat. #7074, Cell Signaling Technology, USA) or anti-mouse IgG (diluted 1:5000, Cat. #7076, Cell Signaling Technology, USA) for 2 h. Finally, the blots were visualized with enhanced chemiluminescence (ECL) reagents (Millipore, USA). The results of this experiment were quantified by gray analysis using Image J software.

### Culture of NP cells

Primary NP cells were freshly isolated as described in our previous studies with minor modification [38]. The NP tissue was dissected and minced, and then digested with 0.75 mg/ml collagenase (Sigma-Aldrich, USA) for 30 min at 37° C. The suspension was filtered (pore size, 100 μm) and resuspended with Dulbecco’s modified Eagle’s medium (DMEM; Gibco, USA) and 20% fetal bovine serum (FBS; Biological Industries, Israel). Subsequently, the cells were plated into 75 cm2 cell culture flasks. After 6-7 days, the cultured primary NP cells were passage into 10 cm cell culture plates (Corning, USA) with DMEM, 10% FBS and antibiotics (Gibco, USA). DDP (Sigma Aldrich, USA) was dissolved in DMF at a concentration of 10 mM.

To simulate cellular senescence, the cells were treated with 2×10^-6^M DDP for 48 hours. RT-qPCR were conducted to validate the optimal concentration and treatment duration (Supplementary Figure 1B, 1C). Subsequently, the cells were treated with 0, 1, 2, 5, 10 or 20 ug/mL Omilancor for 0, 3, 6, 12, 24 or 48 hours to determine the optimal concentration and treatment duration. (Supplementary Figure 1D, 1E).

### Senescence-associated β-galactosidase (SA-β-gal) staining and quantification

Cells were washed to remove drugs and incubated for an additional 3 days in fresh media. *In situ* staining of SA-β-gal with cells or frozen sections were performed using a SA-β-gal staining kit (Beyotime Institute of Biotechnology, China) following the manufacturer’s instructions. Cells were considered positive when the cytoplasm was stained with SA-β-gal. All experiments were performed in triplicate.

### Immunofluorescence

Cells were fixed with 4% paraformaldehyde in PBS for 15 min, after washed gently by PBS, cells were blocked by 10% goat serum (Thermo Fisher) for 1 h. Subsequently, cells were then incubated with MAP2K6 (diluted 1:200, Cat. #12280, Cell Signaling Technology, USA) or p21 (diluted 1:200, Cat. #2947, Cell Signaling Technology, USA) antibody over night at 4° C. The next day, the cells were incubated with fluorescent secondary antibody for 2 h at room temperature, and then counterstained with DAPI. The digital images of cells were taken using a Leica TCS SP8 microscope (Wetzlar, Germany). Fluorescence intensity was measured using ImageJ, and the count data were analyzed using the chi-square test.

### Molecular docking screening and visualization

AutoDock is a suite of free open–source software for the computational docking and virtual screening of small molecules to macromolecular receptors. The structure of MAP2K6 was predicted using AutoDockTools-1.5.6. The model quality was assessed using the predicted aligned error. The molecular dockings and virtual screening of the predicted MAP2K6 and potential ligands structures were simulated and analyzed with AutoDock Vina and AutoLigand, and visualized with PyMOL 2.3.2 (Supplementary Table 2).

### Animal experiment

An IDD rat model was successfully established as described in previous studies [39, 40]. In short, adult Sprague Dawley Rats (SD rats) (Shanghai Laboratory Animal Center, Shanghai, China) weighing approximately 200-250 g at about 10 weeks were used in this experiment. The procedures were approved by the Medical Ethics Committee of Luoyang Central Hospital (Luoyang, China). The rats were randomly divided into three groups: for the AF puncture model, the rats were anesthetized with 1% sodium pentobarbital (50 mg/kg) and placed in the supine position. A midline abdominal incision was made in the front to visualize the L3/4 intervertebral discs. A 27-G needle was inserted into the L3-L4 disc parallel to the endplates by 1.0 mm, rotated in the axial direction by 180° and held for 30 s, and a vessel clamp was used to grip on the marker on the needle to control the depth.

For the Omilancor treatment, Omilancor (BioLegend, USA) was intraperitoneally injected at 4 mg/kg every two days, for a total duration of two weeks before operation.

After 8 weeks, the rats were anesthetized with pentobarbital sodium. After lumbar MRI examinations, discs were harvested for further experiments.

### Statistical analysis

Each experiment is repeated at least three times independently. Data are presented as mean ± standard deviations. Data from multiple groups were compared using One-way analysis of variance (ANOVA). Student’s t test was used to analyze the differences between two groups. Statistical analyses were performed using SPSS software version 24.0 for Windows (IBM, USA). p < 0.05 was considered to indicate statistically significant.

### Data and materials availability

All data of this study are included within the article.

## Supplementary Material

Supplementary Figure 1

Supplementary Table 1

Supplementary Table 2
