# Supplementary material for: Omilancor mitigates the senescence of nucleus pulposus cells induced by DDP through targeting MAP2K6
Source: Aging (Albany NY). 2024 Mar 20;16(6):5050–64. doi: 10.18632/aging.205588 (PMC11006466; doi:10.18632/aging.205588)
Supplement: Supplementary Figure 1 [file aging-16-205588-s001.pdf]

SUPPLEMENTARY FIGURE

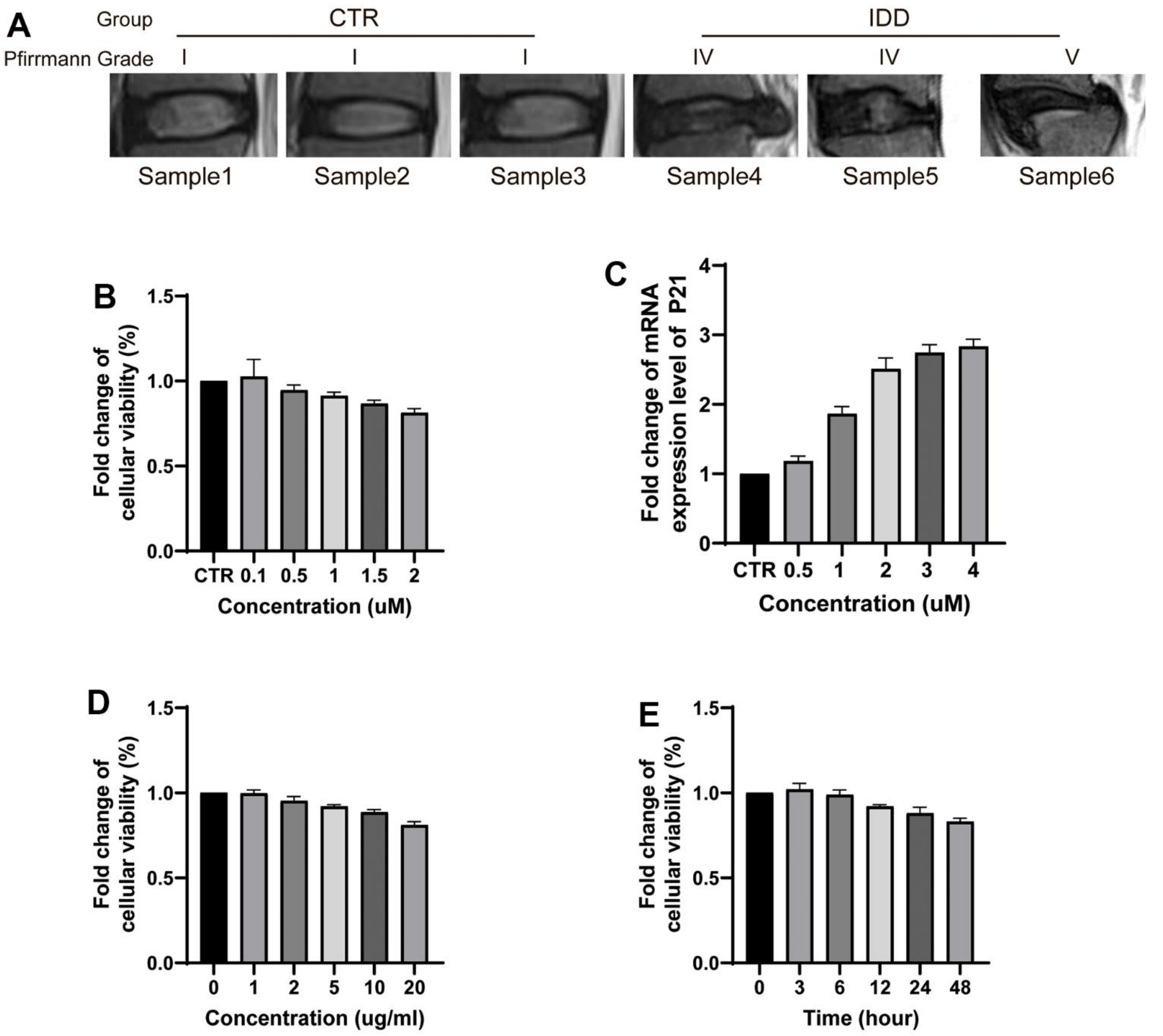

Supplementary Figure 1. Representative T2 image of human IVDs, concentration and time screening of DDP and Omiclancor. (**A**) Representative T2 image of human IVDs in different Pfirrmann grade. (**B**) CCK8 of DDP treatment on NPCs with different concentration. (**C**) RT-qPCR of P21 expression with different concentration of DDP treatment on NPCs. (**D**, **E**) CCK8 of Omiclancor treatment on NPCs with different concentration and time. CTR, control group; IDD, intervertebral disc degeneration group. n = 3 each group. Data are represented as mean ± standard deviation.
